# Effect of Pre-exposure Use of Amantadine on COVID-19 Infection: A Hospital-Based Cohort Study in Patients With Parkinson's Disease or Multiple Sclerosis

**DOI:** 10.3389/fneur.2021.704186

**Published:** 2021-10-07

**Authors:** Walaa A. Kamel, Mohmed I. Kamel, Almunther Alhasawi, Sameh Elmasry, Fajer AlHamdan, Jasem Y. Al-Hashel

**Affiliations:** ^1^Neurology Department, Faculty of Medicine, Beni-Suef University, Beni-Suef, Egypt; ^2^Neurology Department, Ibn-Sina Hospital, Kuwait City, Kuwait; ^3^Occupational and Environmental Medicine, Alexandria University, Alexandria, Egypt; ^4^Internal Medicine and Infectious Diseases Consultant, Infectious Disease Hospital, Kuwait City, Kuwait; ^5^Internal Medicine Department, Al-Sabah Hospital, Kuwait City, Kuwait; ^6^Department of Medicine, Faculty of Medicine, Kuwait University, Kuwait City, Kuwait

**Keywords:** Amantadine, pre-exposure, COVID-19, infection, multiple sclerosis, Parkinson's disease

## Abstract

**Background:** Amantadine has been proposed to inhibit E-channel conductance in reconstituted lipid bilayers of severe acute respiratory syndrome coronavirus 2 (SARS-CoV-2). We aimed to study whether patients on amantadine have altered risks of contracting COVID-19 infection.

**Methods:** We conducted a hospital-based, observational, retrospective cohort study using data for patients on amantadine supported by data given by the patients through an online questionnaire. We included registered amantadine users in our hospital for 6 months or more on March 1, 2020, and non-amantadine users to act as the control group. We used forced entry, multiple logistic regression models to estimate adjusted ORs for amantadine adjusting for the confounders.

**Findings:** Between September 1, 2019, and March 1, 2020, 212 patients with Parkinson's disease (PD) or multiple sclerosis (MS) received greater than one equal to two prescriptions of amantadine. We selected a random sample of diagnoses which matched 424 patients of non-amantadine users (1:2) as a control group (424 patients). Between March 1, 2020, and March 1, 2021, 256 patients responded to our online questionnaire, 87 patients were on amantadine (group I), and 169 patients were not (control group, group II). COVID-19 disease infection proved to be 5.7 and 11.8% in group I and II patients, respectively. Increased odds of COVID-19 in multivariable-adjusted models were associated with old age and history of contact with COVID cases. Amantadine was associated with a significantly reduced risk of COVID-19 disease infection (adjusted OR 0.256, 95% CI 0.074–0.888).

**Interpretation:** Amantadine is associated with a reduced risk of COVID-19 infection after adjusting for a broad range of variables. History of contact with COVID cases and old age are risk factors for COVID-19 infection. Therefore, we recommended randomized clinical trials investigating amantadine use for the prevention of COVID-19.

## Introduction

Antiviral properties for amantadine were initially reported and first used to treat influenza A in 1963 and approved as a prophylactic treatment in 1976 against the influenza virus A (1). Due to the high resistance of amantadine, is not recommended for the treatment of influenza A (2). Amantadine was tried for the treatment of different neurological diseases and approved by the Food Drug Administration (FDA) to treat levodopa-induced dyskinesia (LID) in Parkinson's disease (PD) (3). Off-label uses include improving fatigue in multiple sclerosis (MS) (4) and improving arousal following a brain injury. At the time of the severe acute respiratory syndrome (SARS) epidemic in 2002, amantadine was studied (*in vitro*) and showed therapeutic potential (5).

Recognition of modest antiviral effects shown for adamantanes led to the suggestion that it could be repurposed for COVID-19 (6, 7). This was supported by the fact that patients on this antiviral drug were serologically negative after being in contact with positive cases. Moreover, those who tested positive did not develop the symptoms caused by the virus (8).

Many theories explained the antiviral mechanism as a lipophilic molecule that traverses the lysosome membrane. Amantadine acts as an alkalizing agent that hinders viral ribo nucleic acid (RNA) release into the cell (9).

It was hypothesized that amantadine would give a superior outcome due to decreased infectivity and replication of the virus, based on the fact that amantadine significantly downregulated expression cathepsin L (CTSL) gene coding for the CTSL and lysosomal protease involved in severe acute respiratory syndrome coronavirus 2 (SARS-CoV-2) human cell entry (10). Clinical studies are needed to examine the therapeutic utility of amantadine in COVID-19 infection.

Evaluations of the effectiveness of amantadine use for the prevention of SARS-CoV-2 infection are lacking. In this hospital-based cohort study, we aimed to investigate if ongoing amantadine use in the 6 m before March 1, 2020, considered the start of the outbreak in Kuwait (i.e., analogous to prophylactic use before exposure) was associated with a lesser risk of contracting COVID-19 infection. As a result of understanding the associations between medications and improved outcomes, clinical trials could provide a base for further insights into disease mechanisms, pathogenesis, and possible preventive measures if confirmed from cohort studies.

## Methods

### Data Source and Study Population

**Figure 1:**
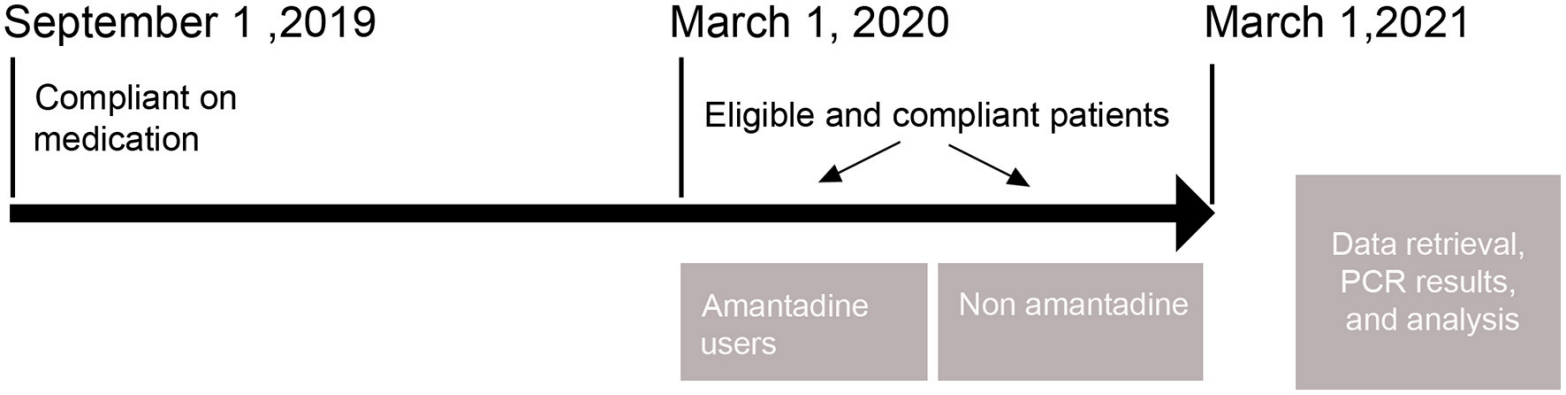
Study diagram.

We conducted a retrospective cohort study using electronic health record data from hospital care practices focusing on patients with the diagnosis of PD and MS who were compliant on their usual medications for at least 6 months before enrollment, with information on age, sex, and medications. Institutional Review Board approval was obtained from our hospital and the ministry of health (Figure 1).

#### Inclusion Criteria

All are 16 years old or above.

#### Patients

We selected participants from Parkinson's disease and multiple sclerosis patients who attended the outpatient clinic. Patients on amantadine acted as a case group and non-amantadine users as a control group.

#### Exclusion Criteria

- Patients who were not regularly on amantadine in the last 6 months before enrollment in the study.
- Recent change in his medications (previous 6 months).
- Patients outside Kuwait during the COVID-19 pandemic period (difficult to get their Polymerase Chain Reaction (PCR) results).
- MS patients who recently (in the past year) received glucocorticoids that may increase the risk of contracting infections (11), including COVID-19 (12).

We divided patients according to their current treatment into two groups; amantadine-user group (group I) (*n* = 212) and a sample of patients with randomly selected diagnoses matched non-amantadine users group II, with the proportion of 1:2.

### Survey Assessments

Participants answered an online questionnaire (created by W. AK.). Surveys queried about; (1) amantadine exposure and dose, (2) COVID-19-related symptoms, (3) testing status and outcomes, (4) comorbid conditions, (5) smoking status, (6) employment, (7) personal and household contact, (8) social distancing practices, (9) mask use, history of travel, (10) COVID-19 infection symptoms (as; fever, respiratory symptoms, catarrhal symptoms), (11) the result of COVID−19 PCR in case of infection, (12) symptoms indicating severe infection (such as; need for O2 use, hospitalization, intensive care unit (ICU) admission, or ventilatory need), and (13) the effect of infection on main neurologic disease, mood, and memory. Results of COVID-19 PCR testing of the respondents were blindly retrieved using the national COVID-19 database (M.WH.).

### Data Collection

We collected data on socio-demographic characteristics, chronic diseases, medications, COVID 19 risk factors, the severity of symptoms, swab results, and sequence of COVID infection on different neurologic symptoms.

### Statistical Analysis

We presented continuous variables as either the means ± standard deviations (SDs) or the medians (with interquartile ranges). For categorical variables, we calculated the frequency rates and percentages of patients in each category. We compared continuous variables using independent group *t*-tests in normally distributed data. Otherwise, we used the Mann–Whitney test. We compared the proportions of categorical variables using the χ^2^-test, although we used Fisher's exact test when the data were limited. Multiple logistic regression analysis was applied to determine the prognostic factors, and the odds ratio (OR) with 95% confidence interval (95% CI) was reported. Statistical analyses were performed using SPSS 21.

## Results

### Overall Study Population

**Figure 2:**
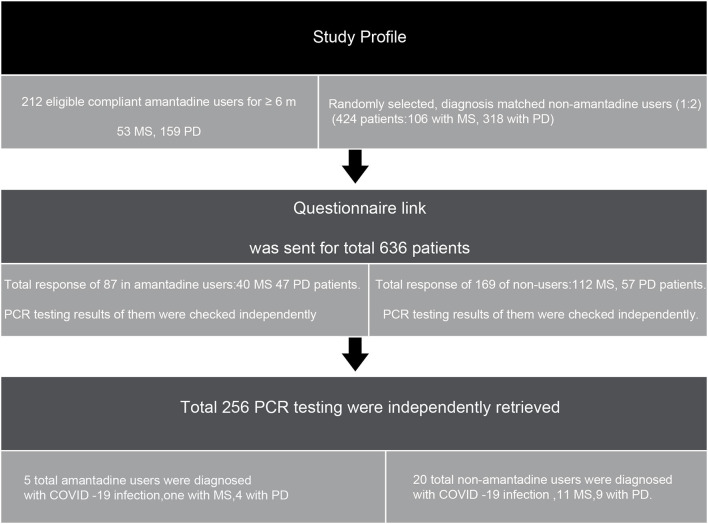
Study profile.

The study design is shown in Figure 1. Two hundred twelve patients were identified (with the diagnosis of PD or MS) to be compliant on amantadine for at least 6 months before March 1, 2020 (i.e., index date). A group of patients with randomly selected diagnoses matched the number of PD, MS patients who were not on amantadine. After sending the questionnaire link to patients, we got 256 responses in both groups (Figures 1, 2).

### Presenting Characteristics

We included 256 patients in our analysis: 87 patients were amantadine users, and 169 were non-users. The results showed that the mean age ± SD of group I was higher than that of group II (52.5 ± 12.4 [31, 75] vs. 46.9 ± 16.2 [16, 84]). Amantadine users are more likely to be men (50.6% of users were women; 56.2% of non-users were women); other demographic characteristics between exposure groups were broadly similar (Table 1).

**Table 1:** Socio-demographic characteristics of both groups.

**Characteristic**	**Group I** **(*N*, %)** **87**	**Group II** **(*N*, %)** **169**	**Total** **(*n* = 256)**	***P***
Parkinson's disease	47 (54)	57 (33.7)	104 (40.6)	
Multiple sclerosis	40 (46)	112 (66.3)	154 (59.4)	
**Age, 10 years**				
20–29	0 (0.0)	22 (13.0)	22 (8.6)	0.003^*^
30–39	14 (16.1)	45 (26.6)	59 (23)	
40–49	23 (26.4)	36 (21.3)	59 (23)	
50–59	23 (26.4)	26 (15.4)	49 (19.1)	
60+	27 (31.0)	40 (23.7)	67 (26.2)	
Mean ± SD	52.48 ± 12.39	46.93 ± 16.18		
	(31, 75)	(16, 84)		
**Gender**				
Male	44 (50.6%)	74 (43.8)	118 (46.1)	0.302
Female	43 (49.4)	95 (56.2)	138 (53.9)	
**Family members**				
1–2	14 (16.1)	21 (12.4)	35 (13.7)	0.097
3–4	24 (27.6)	27 (16.0)	51 (19.9)	
5–6	22 (25.3)	58 (34.3)	80 (31.3)	
7–8	12 (13.8)	20 (11.8)	32 (12.5)	
9+	15 (17.2)	43 (25.4)	58 (22.7)	
**Employment status**				0.369
Not employed	56 (64.4)	99 (58.6)	15 5(60.5)	
Employed	31 (35.6)	70 (41.4)	101 (39.5)	
**Chronic disease**				0.671
No	47 (54.0)	96 (56.8)	96 (56.8)	
Yes	40 (46.0)	73 (43.2)	113 (44.1)	
Hypertension	22 (25.3)	29 (17.2)	51 (19.9)	0.123
DM	18(20.7)	22 (13)	40 (15.6)	0.11
Dyslipidemia	15 (17.2)	15 (8.9)	30 (11.7)	0.049^*^
Heart disease	6 (6.9)	6 (3.6)	12 (4.7)	0.230
Respiratory	5 (5.7)	16 (9.5)	21 (8.2)	0.304
Renal	3 (3.4)	4 (2.4)	7 (2.7)	0.615
Hepatic	2 (2.3)	2 (1.2)	4 (1.6)	0.495
Other	13 (14.9)	25 (14.8)	38 (14.8)	0.975
**Obesity (yes)**	5 (5.7)	10 (5.9)	15 (5.9)	0.956
**Current smoker (yes)**	15 (17.2)	42 (24.9)	57 (22.3)	0.166

* *Significant, P < 0.05. The bold values indicates statistically significant*.

The amantadine users group had more patients with comorbidities; 46% of them have a chronic illness compared to 43.2% in the control group. Hypertension (25.3 vs. 17.2%), diabetes (20.7 vs. 13%), heart disease (6.9 vs. 3.6%), dyslipidemia (17.2 vs. 8.9%), and chronic kidney disease (3.4 vs. 2.4%). The clinical data listed in Table 1.

### Amantadine Dose in Group I

Amantadine dose ranges from 100 mg daily to 400 mg, with no statistically significant difference in the dose between patients infected with COVID compared with those who were not. Dose mean, SD (2.1 ± 0.9 vs. 2.2 ± 1.3) (Table 2).

**Table 2:** Amantadine doses in group I.

**Amantadine**	**Daily dose (mg)**	**COVID-19**		**Total**
		**NO (*N*, %)**	**Yes (*N*, %)**	
	100	21 (25.6)	2 (40.0)	23 (26.4)
	200	37 (45.1)	1 (20.0)	38 (43.7)
	300	19 (23.2)	1 (20.0)	20 (23.0)
	400	5 (6.1)	1 (20.0)	6 (6.9)
**Total**		82 (100)	5 (100)	87 (100)
Mean ± SD		2.1 ± 0.9	2.2 ± 1.3	

*t = 0.252, P = 0.801*.

### COVID-19 Infection and Related Risk Factors

Regarding the COVID-19 symptoms, almost the same percentage of patients in both groups have symptoms suggestive of COVID-19 infection (18.9 vs. 18.4%). 40.2% of the total number of patients in group I underwent PCR testing for COVID-19(5.7% of them have positive results) in comparison to 44.3% of patients in group II (11.8% of them resulted as positive) (Table 3).

**Table 3:** COVID-19 risk factors, symptoms and their severity, and PCR results.

	**Group I** ***N* (%)**	**Group II** ***N* (%)**	***P***
**Adopting precautions**			0.671
No	13 (14.9)	22 (13.0)	
Yes	74 (85.1)	147 (87.0)	
**History of travel**			0.932
No	79 (90.8)	154 (91.1)	
Yes	8 (9.2)	15 (8.9)	
**Contact with a case**			0.469
No	65 (74.7)	119 (70.4)	
Yes	22 (25.3)	50 (29.6)	
**COVID-19 symptoms**			0.916
No	71 (81.6)	137 (81.1)	
Yes	16 (18.4)	32 (18.9)	
**Swab for SARS CoV2**			0.299
Not done	52 (59.8)	94 (55.6)	
Negative	30 (34.5)	55 (32.5)	
Positive	5 (5.7)	20 (11.8)	
Symptomatic	5 (100.0)	17 (85.0)	1.00
Hospitalized	2 (40.0)	5 (25.0)	0.597
ICU	0 (0.0)	1 (5.0)	1.00
Ventilation	0 (0.0)	1 (5.0)	1.00
Oxygen use	1 (20.0)	7 (35.0)	0.643

There was no statistically significant difference between both groups regarding different known risk factors of COVID-19 infection (contact with a case, history of travel, Adopting precautions) (Table 3).

Between March 1, 2020, and March 1, 2021, there were 25 COVID-19 infections (diagnosed by PCR testing) among people with PD or MS, 5 (20%) of which were among regular users of amantadine. Severe symptoms in the form of (hospitalized, needed ICU, those on a ventilator, needed oxygen most of the time) were noted more in the non-users, although not reaching a statistical significance. The clinical data showed in Table 3.

### Post COVID Infection Period

Worsening of the baseline neurological disease was noted in 45% of positive patients in group II, with no worsening noted among COVID patients in amantadine users. However, this difference does not reach a statistical significance. Furthermore, we reported worsened memory and mood in both groups, with no statistically significant difference between the two groups (Table 4).

**Table 4:** Sequence of COVID infection on different neurologic symptoms.

**Effect of COVID infection**	**Group I**	**Group II**	***P***
Worsening of baseline neurological disease	0 (0.0)	9 (45.0)	0.123
Worsened memory	1 (20.0)	7 (35.0)	1.00
Worsened mood	2 (40.0)	11 (55.0)	0.645

### Associations of Outcome With Amantadine Exposure

#### Risk Factors for COVID-19 Infection

Older age and history of contact with COVID case were each associated with increased odds of COVID-19 in multivariable-adjusted models as follows: (OR: 1.048; 95% CI: 1.005, 1.092), (OR: 17.025; 95% CI 5.348, 54.199), respectively (Table 5).

**Table 5:** Odds ratio and its 95% confidence limits of COVID-19 predictors using forced entry multiple logistic regression model.

**Predictor**	**β**	***P***	**OR**	**95% CI of OR**
Age by 10 y	0.047	0.028^*^	1.048	1.005, 1.092
Gender	−0.237	0.665	0.789	0.270, 2.304
Nationality	0.406	0.491	1.501	0.472, 4.766
Family members	0.079	0.171	1.082	0.967, 1.211
Work	0.237	0.671	1.267	0.424, 3.786
Smoking	−0.127	0.843	0.881	0.250, 3.100
Chronic disease	−1.963	0.003^*^	0.140	0.038, 0.516
Taking precautions	−1.781	0.006^*^	0.168	0.047, 0.598
History of contact	2.835	<0.001^*^	17.025	5.348, 54.199
Using amantadine	−1.362	0.032^*^	0.256	0.074, 0.888
Constant	−4.107	0.005^*^	–	–

* *Significant, P < 0.05. The bold values indicates statistically significant*.

Amantadine was associated with a significantly reduced risk of COVID-19 disease infection after adjustment for several covariates, including age, sex, and other risk factors (adjusted OR 0.256, 95% CI 0.074–0.888). Similarly, taking precautions was associated with a significantly reduced risk of COVID-19 disease (OR: 0.168; 95% CI: 0.047, 0.598) (Table 5).

## Discussion

### Summary of Key Results

In our hospital-based retrospective study, we assessed risk factors for COVID-19 among a cohort of patients known to have PD or MS and its relation to the use of amantadine.

Consistent with existing evidence, individuals who have a history of contact with COVID-19 cases had a higher risk of being diagnosed with SARS-CoV-2 (13). In addition, older people have a higher COVID-19 risk (14). Amantadine prescriptions were associated with a reduced risk of COVID-19 PCR positive cases adjusted for a wide area of demographic factors, potential comorbidities, and other medication. We found a shred of evidence that pre-exposure use of amantadine was associated with a lower risk of COVID-19 infection.

### Comparisons With the Literature

Several studies assessed the anti-inflammatory effects of amantadine (15, 16). However, to date, published data reported associations between chronic medication with amantadine and COVID-19 infections are limited to clinical observations. In this respect, a preliminary report by Rejdak and Grieb involved 15 patients with PD, MS with previous exposure to amantadine at least 3 months previous to involvement in the study. All these amantadine users had contact with confirmed COVID-19 patients. However, they remained asymptomatic even after they had tested positive for coronavirus (8). Another observational open-label study involving 15 patients supported the protective theory of amantadine, recently performed by Aranda-Abreu et al. (17), who treated patients infected with SARS-CoV-2 using it for 14 days. The presence of clinical symptoms and positivity for IgG antibodies and negativity of IgM against SARS-CoV-2 at the end of the treatment confirmed the diagnosis of SARS-CoV-2 patients infection. Although these patients recovered successfully, treated with other medications along with amantadine. The proposed mechanism is that amantadine can cross the lysosome membrane as a lipophilic molecule and act as an alkalizing agent which will prevent the release of viral RNA into the cell (10). To assess the severity of COVID-19 in Patients suffering from MS and PD, the authors carried out a questionnaire-based study. None of them (PD, *n* = 5; MS, *n* = 10) developed severe clinical manifestations of infectious disease (18). Similarly, in a Community-Based Case-Control study, 8.5% of PD patients were positive for COVID-19 not medicated with amantadine (19). A case of a 75-year-old woman with PD treated with amantadine and was in direct contact with a positive COVID-19 subject had not had any symptoms related to COVID-19 (20).

Our study has several important strengths. The patient's exposure to medication was not only based on issued prescriptions but was confirmed by asking the patient about actual use, not the one prescribed. Importantly, PCR's results were retrieved blindly by an author without access to patients' questionnaires. The long duration of compliance on medication besides retrieval of one-year data is remarkable to strengths as well.

## Limitations

Several limitations of our study are worth noting. The main is the small cohort of COVID-19 patients. Detailed information on the exact timing of COVID-19 infection is lacking, so we could not incorporate this information into the analyses or assess potential time-varying risk factors or confounding factors.

We have not investigated the relationship between the duration of exposure and the risk of disease in this analysis. There will be an underestimation of total COVID-19 cases (several patients in both groups were not tested for COVID-19) due to the absence of a systematic testing strategy at the start of the pandemic, and a risk of false-negative results as health policy during the study period restricted testing to only COVID-19 patients with severe symptoms or patients before hospitalization for non-COVID-19 indications even in the absence of symptoms suggestive of COVID-19 infection. So, performing a swab or presence of manifestations alone is not a sure marker of suspecting COVID-19 infection and may result in selection bias.

Another limitation is the risk of residual confounding by the use of other medications. In addition, we could not assess the severity of neurological disease that may affect the patient's ability to move and communicate with people; that estimate could be a potential bias. Another important consideration is the potential for exposure misclassification. As with any observational study that uses prescription and dispensed medications' data, we could not accurately capture whether patients took their medicine, causing a potential bias toward null findings. However, we defined exposure based on repeat prescriptions, which is likely to be a good sign of compliance.

## Data Availability Statement

The raw data supporting the conclusions of this article will be made available by the authors upon reasonable request.

## Ethics Statement

The studies involving human participants were reviewed and approved by the ministry of health IRB, Kuwait. Written informed consent for participation was not required for this study in accordance with the national legislation and the institutional requirements.

## Author Contributions

WK: design, questionnaire development, and manuscript write-up. AA: retrieved PCR details and helped in manuscript write-up. JA-H: supervised the project. SE: study idea and theory development. MK: statistical analysis. FA: data collection. All authors contributed to the article and approved the submitted version.

## Conflict of Interest

The authors declare that the research was conducted in the absence of any commercial or financial relationships that could be construed as a potential conflict of interest.

## Publisher's Note

All claims expressed in this article are solely those of the authors and do not necessarily represent those of their affiliated organizations, or those of the publisher, the editors and the reviewers. Any product that may be evaluated in this article, or claim that may be made by its manufacturer, is not guaranteed or endorsed by the publisher.

## Abbreviations

SARS-CoV-2: severe acute respiratory syndrome coronavirus 2
PD: Parkinson's disease
MS: multiple sclerosis
FDA: food drug administration
LID: levodopa-induced dyskinesia
RNA: ribo nucleic acid
CTSL: cathepsin L
PCR: polymerase chain reaction
ICU: intensive care unit
SD: standard deviations
HR: hazard ratio
CI: confidence interval
OR: odds ratio
PCR: polymerase chain reaction.
